# Compressive Sensing via Nonlocal Smoothed Rank Function

**DOI:** 10.1371/journal.pone.0162041

**Published:** 2016-09-01

**Authors:** Ya-Ru Fan, Ting-Zhu Huang, Jun Liu, Xi-Le Zhao

**Affiliations:** 1 School of Mathematical Sciences/Research Center for Image and Vision Computing, University of Electronic Science and Technology, Chengdu, Sichuan, 611731, P. R. China; 2 School of Mathematics and Statistics, Northeast Normal University, Changchun, Jilin, 130024, P. R. China; University of North Carolina at Chapel Hill, UNITED STATES

## Abstract

Compressive sensing (CS) theory asserts that we can reconstruct signals and images with only a small number of samples or measurements. Recent works exploiting the nonlocal similarity have led to better results in various CS studies. To better exploit the nonlocal similarity, in this paper, we propose a non-convex smoothed rank function based model for CS image reconstruction. We also propose an efficient alternating minimization method to solve the proposed model, which reduces a difficult and coupled problem to two tractable subproblems. Experimental results have shown that the proposed method performs better than several existing state-of-the-art CS methods for image reconstruction.

## Introduction

Compressive sensing (CS) [1, 2] allows us to reconstruct high dimensional data with only a small number of samples or measurements, and captures only useful information and has the potential of significantly improving the energy efficiency of sensors in the real-world applications. The key idea behind CS is that the majority of real-world signals including images and videos, can be sparsely represented by given some appropriate basis. Due to the positive theoretical and experimental results, many CS-based imaging methods have been proposed and applied to the various areas such as magnetic resonance imaging [3, 4] in medicine, compressed spectral and hyperspectral imaging [5, 6] in industry, neural network [7] in biotechnology.

CS also makes it possible to well restore corrupted signals at a fast speed and the small memory cost. Conventional CS recovery uses ℓ_1_ norm to characterize the sparsity of a signal, and the resulting convex optimization problems are tractable. Although there are several methods can be used to efficiently solve the ℓ_1_ regularization based model for signal recovery [8–10], they only achieve suboptimal recovery performance due to their relaxation of the ℓ_0_ norm based sparse optimization [11]. More recently, structured or group sparsity based methods [12–14] and nonlocal sparsity based methods [15, 16] have provided better results for CS recovery. Intuitively, the structured or group sparsity can reduce the degrees of freedom in the solution, and the nonlocal sparsity explicitly exploits self-similarities of the signal, thereby obtaining more accurate recovery performance than the common sparsity.

In CS studies, a number of works have suggested that non-convex optimization based approach often yields better results than convex ones though costing higher computational complexity. Therefore, we propose a CS recovery model by considering a non-convex smoothed function to approximate the rank, denoted as smoothed rank function (SRF), as a low-rank regularization. The emphasis behind the proposed model is the utilization of nonlocal sparsity by image patch grouping. Concretely, we group a set of similar image patches to form a matrix **X**_*i*_ for *i*-th exemplar image patch, which is extracted from the test image. Then the matrix **X**_*i*_ is low-rank since similar patches have similar structure. To solve the low-rank optimization problem, we minimize the SRF function to approximate the rank minimization problem, and the resulting problem is a non-convex optimization problem. In order to avoid getting trapped in local solutions, we initialize a rough approximation of the rank, and gradually improve the approximation as the iteration proceeds.

The basic idea of using nonlocal sparsity for image patches has already been used in [16–18] with the very impressive results. There have been an abundant research literatures using the patch-based low-rank as plenty with nonlocal sparsity [19–22]. In [20, 21], the low-rank problem is solved by minimizing the nuclear norm of the low-rank matrix, which leads to a convex minimization problem with many efficient methods available. In [22], a more accurate approximation for rank is proposed by exploiting the logdet function, which can be derived as a weighted nuclear norm. Specifically, compared with previous surrogates for the rank such as nuclear norm and weighted nuclear norm, our surrogate is differentiable and can approximate the rank adaptively.

The outline of this paper is organized as follows. Firstly, we briefly review the background of CS. Secondly, the SRF function is introduced and the model based on SRF for CS image reconstruction is proposed. Thirdly, the optimization algorithm is presented to solve the proposed model. To demonstrate the effectiveness of the proposed method, we show some numerical results on several test images in the following section. In the end, the conclusion and future work are given.

## Background

The CS recovery problem aims to find the sparsest solution $x\in R^{N}$ from the underdetermined linear system **y** = Φ **x**, where $y\in R^{M}$ is the measurements and $\Phi \in R^{M\times N}$, *M* < *N* is the measurement matrix. It can be formulated as follows:

$$\min_{x}{\left||x|\right|}_{0}\;s.t.\;y=\Phi x,$$ (1)

where ||⋅||_0_ is the ℓ_0_ norm counting the number of nonzero elements of **x**. In practical applications, such as signal reconstruction problem, the measurement noise is unavoidable. Then the noisy CS recovery problem is formulated as

$$\min_{x}{\left||x|\right|}_{0}\;s.t.\;||y-{\Phi x||}_{2}\leq \epsilon ,$$ (2)

where ||⋅||_2_ is the ℓ_2_ norm and *ϵ* is the residual error. However, since problem (2) is NP-hard, it is infeasible to solve it directly. Thus it is proposed to replace the ℓ_0_ norm by the convex ℓ_1_ norm, namely

$$\min_{x}{\left||x|\right|}_{1}\;s.t.\;||y-{\Phi x||}_{2}\leq \epsilon ,$$ (3)

which is the well-known basis pursuit problem [2]. The problem (3) is the convex relaxation of problem (2) and easy to solve by some methods including iterative shrinkage algorithm [8], alternating direction method of multipliers (ADMM) [23] and Bregman split algorithm [24]. Although problems (2) and (3) are fundamentally different, they return the same solution in many interesting situations [25]. By using an appropriate regularization parameter *λ*, we can equivalently rewrite the problem (3) as follows:

$$\min_{x}\;||y-{\Phi x||}_{2}^{2}+{\lambda ||x||}_{1}.$$ (4)

By replacing the ℓ_1_ norm by the non-convex ℓ_*p*_ (0 > *p* > 1) norm, in [26], the non-convex optimization problem based on ℓ_*p*_ norm can achieve the more exact CS reconstruction result than the convex one based on the ℓ_1_ norm. Moreover, it has been shown in [18, 27] that the nonlocal sparsity exploiting the self-similarity of the natural image leads to the state-of-the-art performance. The nonlocal sparsity is a significant prior for CS recovery. In this work, we will propose a non-convex CS image recovery approach, which exploits the nonlocal sparsity.

## Nonlocal smoothed rank function

In this section, we present a procedure of patch grouping that uses image self-similarity and leads to the low-rank problem. Hence one underlying assumption is the image exhibits abundant self-similarities. For a test image, we select some exemplar patches of size $\sqrt{n}\times \sqrt{n}$, reordered into the column vector lexicographically and denoted as ${\hat{x}}_{i}\in R^{n}$. For each exemplar patch, the underlying assumption makes it possible to find a number of similar patches. Specially, we employ a variant of k-nearest-neighbor search in a local window for each exemplar patch ${\hat{x}}_{i}$ to find its similar patches as follows:

$$G_{i}=\{i_{j}|\left|\right|{\hat{x}}_{i}-{\hat{x}}_{i_{j}}{\left|\right|}_{2}<c\},$$ (5)

where *c* is a pre-defined threshold and **G**_*i*_ indexes the positions of corresponding similar patches. After patch grouping, we aline the similar patches as column vectors to form a matrix $X_{i}=\left[{\hat{x}}_{i},{\hat{x}}_{i_{1}},\ldots ,{\hat{x}}_{i_{m-1}}\right]$, $X_{i}\in R^{n\times m}$ for the *i*-th exemplar patch ${\hat{x}}_{i}$. Under the above assumption, each matrix **X**_*i*_ has the low-rank property since the similar patches have the similar structures. In this way, the nonlocal sparsity leads to a rank minimization problem in our image reconstruction approach.

Since the resulting matrix **X**_*i*_ also contains the noise during patch grouping, in order to obtain a clean and clear image to well match the ground truth, let **X**_*i*_ = **L**_*i*_+**W**_*i*_, where **L**_*i*_ denotes the low-rank matrix and **W**_*i*_ represents the noise matrix. We can obtain the low-rank matrix **L**_*i*_ by solving the following problem:

$$\min_{L_{i}}\text{rank}\left(L_{i}\right)\;s.t.\;||X_{i}-L_{i}{\left|\right|}_{F}^{2}\leq \epsilon^{2},$$ (6)

where ||⋅||_*F*_ is the Frobenius norm and *ϵ* is the residual error. Unfortunately, problem (6) is NP-hard. A popular heuristic method is to adopt the nuclear norm (sum of the singular values) to replace the rank, i.e.,

$$\min_{L_{i}}\left|\right|L_{i}{\left|\right|}_{*}\;s.t.\;||X_{i}-L_{i}{\left|\right|}_{F}^{2}\leq \epsilon^{2},$$ (7)

where ||⋅||_*_ is the nuclear norm. The problem (7) is a convex surrogate of the rank and can be solved efficiently by the singular value thresholding (SVT) algorithm [28]. However, it is in practice suboptimal due to equally treating each singular value. In [19], Gu et al. have demonstrated that non-convex low-rank approximations adaptively treating the singular values at different scales yield better results than those convex ones.

**Fig 1:**
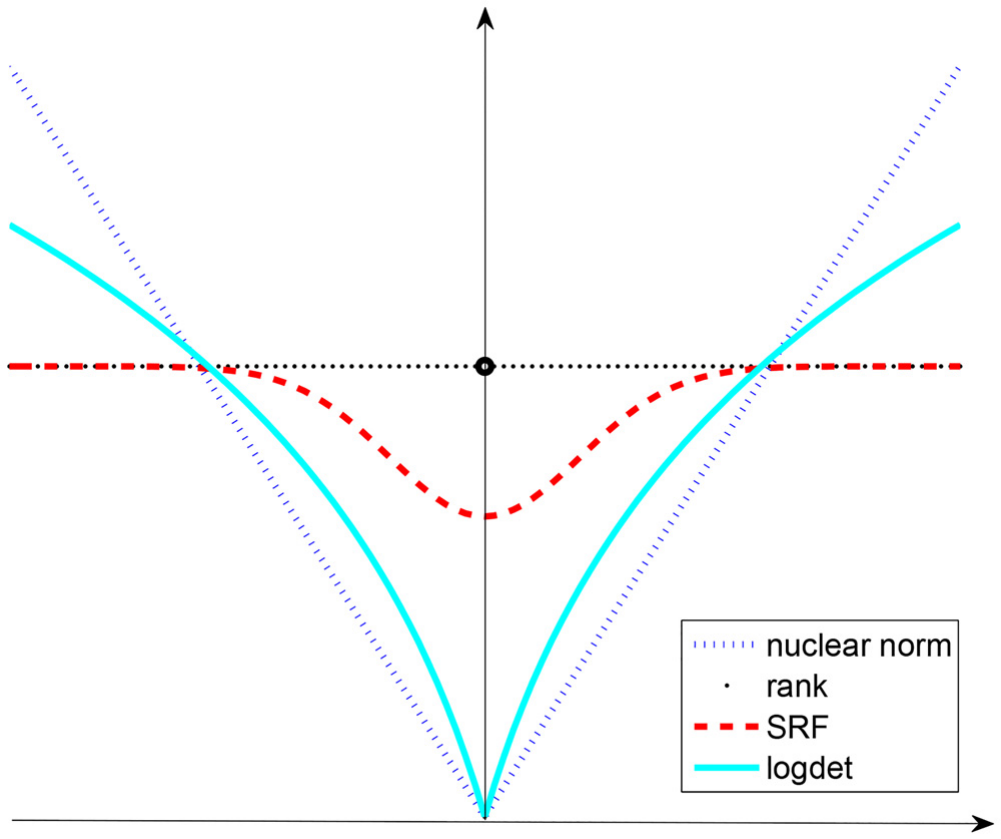
Performance of SRF = *G*_*δ*_(*x*), *rank* = ||*x*||_0_, the nuclear norm = ||*x*||_1_ and *logdet* = log(|*x*|+*ε*) in the case of a scalar, where *δ* = 1 and *ε* = 1.

In [29, 30], they have been shown that for a low-rank matrix $X\in R^{n\times m}$, the rank can be approximated by the following SRF function:

$$G_{\delta }\left(X\right)=\ell -\sum_{j=1}^{\ell }e^{-\sigma_{j}^{2}\left(X\right)/2\delta^{2}},$$ (8)

where *σ*_*j*_(**X**) is the *j*-th singular value of **X**, ℓ = min{*n*, *m*} and *δ* is an adjustable parameter. Although the problem of minimizing *G*_*δ*_(⋅) function using a small *δ* will lead to many local minima, the solution of minimizing *G*_*δ*_(⋅) function converges to the minimum rank solution as *δ* goes to zero [30] as the red curve shown in Fig 1. In order to avoid trapping in local solutions, we initialize a large *δ* and gradually decrease *δ* to improve the degree of approximation for the rank. With the decreasing *δ*, the rank can be better approximated by SRF Eq (8). This technique refines the minimizer of the SRF minimization problem by considering the minimizer of the previous iteration (large *δ*) as the new initial point of current iteration (small *δ*), which makes the solution of the SRF minimization problem get closer to the minimum rank solution during the iterations. Fig 1 intuitively illustrates the comparison of SRF, the rank, the nuclear norm and logdet function in the case of a scalar. We observe that SRF can better approximate the rank than the nuclear norm and logdet function. Therefore, we speculate that the problem of minimizing SRF toward rank minimization problem could achieve better performance. Then, we consider the SRF function as the low-rank regularization for CS image recovery.

For the low-rank matrix **L**_*i*_, the rank minimization problem can be replaced to

$$\min_{L_{i}}G_{\delta }\left(L_{i}\right)\;s.t.\;||X_{i}-L_{i}{\left|\right|}_{F}^{2}\leq \epsilon^{2},$$ (9)

which can be reformulated with an appropriate parameter *λ*,

$$L_{i}=\arg\min_{L_{i}}\frac{1}{2}\left|\right|X_{i}-L_{i}{\left|\right|}_{F}^{2}+\lambda G_{\delta }\left(L_{i}\right).$$ (10)

Obviously, the problem (10) is smoothed and differentiable, and we can adopt the gradient descent method to solve it. For each matrix **X**_*i*_, the same method can be used to obtain the corresponding low-rank matrix **L**_*i*_.

For CS image reconstruction problem, based on above presented *G*_*δ*_(**L**_*i*_), we propose the global model as follows:

$$\left(\hat{x},{\hat{L}}_{i}\right)=\arg\min_{x,L_{i}}||y-{\Phi x||}_{2}^{2}+\eta \sum_{i}\left\{|\right|P_{i}x-L_{i}{\left|\right|}_{F}^{2}+\lambda G_{\delta }\left(L_{i}\right)\},$$ (11)

where *η* is a regularization parameter and **P**_*i*_ **x** = [**P**_*i*_0__ **x**,**P**_*i*_1__ **x**, ⋯,**P**_*i*_*m* − 1__ **x**] is the matrix constituted by the set of similar patches for each exemplar patch **x**_*i*_. The model (11) exploits the nonlocal sparsity of the image patches and non-convexity of the SRF function *G*_*δ*_(⋅). Therefore, we conjecture the proposed method can achieve the good performance. To solve the model (11), we develop an efficient alternative minimization method in the next section.

## Optimization algorithm

It is difficult to directly solve the global model (11) since **x** and **L**_*i*_ is coupled. We use the alternative minimization method to decouple **x** and **L**_*i*_ as follows:

- For **L**_*i*_-subproblem, its optimization problem is
  $${\hat{L}}_{i}=\arg\min_{L_{i}}\left|\right|P_{i}x-L_{i}{\left|\right|}_{F}^{2}+\lambda G_{\delta }\left(L_{i}\right).$$ (12)
- For **x**-subproblem, its optimization problem is
  $$\hat{x}=\arg\min_{x}||y-{\Phi x||}_{2}^{2}+\eta \sum_{i}\left|\right|P_{i}x-L_{i}{\left|\right|}_{F}^{2}.$$ (13)

In the following subsections we will present the optimization algorithm to solve subproblems Eqs (12) and (13) in detail.

### Low-rank matrix optimization algorithm

Let $f\left(L_{i}\right)=\left|\right|P_{i}x-L_{i}{\left|\right|}_{F}^{2}+\lambda G_{\delta }\left(L_{i}\right)$. Then the low-rank matrix **L**_*i*_ can be obtained via the gradient descent method, i.e., $L_{i}^{\left(k+1\right)}\leftarrow L_{i}^{\left(k\right)}-\mu^{\left(k\right)}\nabla f\left(L_{i}^{\left(k\right)}\right)$ where *μ*^(*k*)^ is the step size in the *k*-th iteration and ∇*f*(⋅) is the gradient of *f*(⋅). Before deriving ∇*f*(⋅), we show the gradient of SRF *G*_*δ*_(**L**_*i*_) at **L**_*i*_ [30] as follows:

$$\nabla G_{\delta }\left(L_{i}\right)=-Udiag\left(-\frac{\sigma_{1}}{\delta^{2}}e^{-\sigma_{1}^{2}/2\delta^{2}},\cdots ,-\frac{\sigma_{\ell }}{\delta^{2}}e^{-\sigma_{\ell }^{2}/2\delta^{2}}\right)V^{T},$$ (14)

where matrixes **U** and **V** and singular values *σ*_*i*_ (*i* = 1, ⋯, ℓ) come from the singular value decomposition (SVD) of **L**_*i*_, which is obtained in previous iteration,

$$L_{i}=Udiag\left(\sigma_{1},\cdots ,\sigma_{\ell }\right)V^{T}.$$ (15)

Then, it is easy to derive the gradient of the function *f*(**L**_*i*_),

$$\nabla f\left(L_{i}\right)=-2\left(P_{i}x-L_{i}\right)-\lambda Udiag\left(-\frac{\sigma_{1}}{\delta^{2}}e^{-\sigma_{1}^{2}/2\delta^{2}},\cdots ,-\frac{\sigma_{\ell }}{\delta^{2}}e^{-\sigma_{\ell }^{2}/2\delta^{2}}\right)V^{T}.$$ (16)

According to the gradient descent method, at each iteration, one has

$$L_{i}\leftarrow L_{i}-\mu \nabla f\left(L_{i}\right).$$ (17)

In addition, the step size *μ* in formula (17) should be set in a decreasing order to return a better result. Following the papers [30, 31], we set *μ* = *δ*^2^ to decrease *μ* proportional to *δ*^2^. Then, above formula (17) incorporating the Eq (16) can be rewritten as

$$L_{i}\leftarrow \xi L_{i}+\left(1-\xi \right)P_{i}x-\lambda Udiag\left(\sigma_{1}e^{-\sigma_{1}^{2}/2\delta^{2}},\cdots ,\sigma_{\ell }e^{-\sigma_{\ell }^{2}/2\delta^{2}}\right)V^{T},$$ (18)

where *ξ* = 1 − 2*δ*^2^.

### Image reconstruction via alternating direction method of multipliers

After obtaining the low-rank matrix **L**_*i*_, we reconstruct the image **x** via solving the problem (13). It is clear that the problem (13) is a quadratic optimization problem with a closed-form solution,

$$x={\left(\Phi^{H}\Phi +\eta \sum_{i}P_{i}^{T}P_{i}\right)}^{-1}\left(\Phi^{H}y+\eta \sum_{i}P_{i}^{T}L_{i}\right).$$ (19)

However, the inverse of the matrix $\left(\Phi^{H}\Phi +\eta \sum_{i}P_{i}^{T}P_{i}\right)$ is very large and difficult to compute. Therefore, we consider to reconstruct the image **x** in the framework of ADMM, which also leads to the closed-form solutions for each subproblem. The ADMM method is often used to image restoration [32–36].

Based on the definition of ADMM, we present the augmented Lagrangian function of the problem (13) as follows:

$$L_{d}\left(x,z\right)=||y-{\Phi x||}_{2}^{2}+\beta ||x-z+\frac{d}{2\beta }{\left|\right|}_{2}^{2}+\eta \sum_{i}\left|\right|P_{i}z-L_{i}{\left|\right|}_{F}^{2},$$ (20)

where **z** = **x** is the auxiliary variable, **d** is the Lagrangian multiplier, and *β* > 0 is a scalar. With respect to the variables **x**, **z** and **d**, they are decoupled in the framework of ADMM, thus, can be solved separately, leading to the following iterations:

$$z^{\left(k+1\right)}=\arg\min_{z}\beta ||x^{\left(k\right)}-z+\frac{d^{\left(k\right)}}{2\beta }{\left|\right|}_{2}^{2}+\eta \sum_{i}\left|\right|P_{i}z-L_{i}{\left|\right|}_{F}^{2},$$ (21)

$$x^{\left(k+1\right)}=\arg\min_{x}||y-{\Phi x||}_{2}^{2}+\beta ||x^{\left(k\right)}-z^{\left(k+1\right)}+\frac{d^{\left(k\right)}}{2\beta }{\left|\right|}_{2}^{2},$$ (22)

$$d^{\left(k+1\right)}=d^{\left(k\right)}+\beta \left(x^{\left(k+1\right)}-z^{\left(k+1\right)}\right).$$ (23)

Clearly, both subproblems Eqs (21) and (22) are quadratic optimization problem and have the closed-form solutions. For the subproblem Eq (21), its explicit solution is:

$$z^{\left(k+1\right)}={\left(\eta \sum_{i}P_{i}^{T}P_{i}+\beta I\right)}^{-1}\left(\beta x^{\left(k\right)}+\frac{d^{\left(k\right)}}{2}+\eta \sum_{i}P_{i}^{T}L_{i}\right).$$ (24)

For the subproblem Eq (22), according to its first-order derivation for **x**, we can derive the following equation:

$$\left(\Phi^{H}\Phi +\beta I\right)x^{\left(k+1\right)}=\Phi^{H}y+\beta z^{\left(k+1\right)}-\frac{d^{\left(k\right)}}{2}.$$ (25)

Considering measurement matrix Φ is a partial Fourier transform matrix, we can transform above problem from image space to Fourier space to efficiently obtain **x**. Concretely, we let Φ = **DF**, where **D** is the down-sampling matrix and **F** is the fourier transform matrix. It is easy to achieve

$$F\left({\left(DF\right)}^{H}DF+\beta I\right)F^{H}Fx^{\left(k+1\right)}=F{\left(DF\right)}^{H}y+F\left(\beta z^{\left(k+1\right)}-\frac{d^{\left(k\right)}}{2}\right),$$ (26)

and then **x**^(*k*+1)^ can be drawn

$$x^{\left(k+1\right)}=F^{H}{\left(D^{T}D+\beta I\right)}^{-1}\left(D^{T}y+F\left(\beta z^{\left(k+1\right)}-\frac{d^{\left(k\right)}}{2}\right)\right).$$ (27)

We simultaneously obtain the low-rank matrix **L**_*i*_ and the image **x** by the alternative minimization method, and the overall procedure is summarized below as **Algorithm 1**. Empirically we have found that **Algorithm 1** is convergent, but in theory the convergence analysis of **Algorithm 1** is difficult to give due to the non-convex subproblem. Although there are some papers have proved the convergence of their non-convex optimization problem, these proofs hold in a few unrealistic and rigorous assumptions. Specially, to save computational complexity, we set *J* = *K* = 1 in **Algorithm 1**. Moreover, we estimate an image $\hat{x}$ using the discrete cosine transform (DCT) method as the initial solution for a better initial point, which has been seen in [22]. As iteration increases, the high accuracy results will be achieved.

The complexity of **Algorithm 1** is *O*([*T*_*s*_ + log *N*]*N*) (*N* is the total number of image pixels and *T*_*s*_ is the average complexity to compute similar patches per exemplar patch), which is mainly generated by the DCT method in 1 step of initialization and the Fourier transform in 4 (b) step of inner loop. The complexity of SVD in 3 (a) step of inner loop, i.e. *O*(*n* × *m*^2^), can be ignored due to *n*, *m* ≪ *N*. Therefore, the proposed method is practical feasible and promising.

**Algorithm 1** CS via the SRF function

**Initialization**:

1. Estimate an initial image $\hat{x}$ using the discrete cosine transform (DCT) method;

2. Set regularization parameters *λ* and *η*, and parameter *β* > 0;

3. Let $\delta^{\left(0\right)}=\bar{\delta }$, *μ*^(0)^ = (*δ*^(0)^)^2^, $x^{\left(1\right)}=\hat{x}$, **d**^(1)^ = 0;

4. Find a set of similar patches by the method Eq (5) for each exemplar patch using **x**^(1)^;

**Outer loop**: for *s* = 1, 2, …, *S* do

1. Match similar patches into a matrix **X**_*i*_ for each exemplar patch using **x**^(*s*)^;

2. Set $L_{i}^{\left(0\right)}=X_{i}$;

3.**Inner loop** (solving the **L**_*i*_-subproblem Eq (12)): for *j* = 1, 2, …, *J* do

 (a) Compute the SVD of $L_{i}^{\left(j-1\right)}=Udiag\left(\sigma_{1},\ldots ,\sigma_{\ell }\right)V^{H}$;

 (b) Compute $\nabla f(L_{i}^{\left(j-1\right)})$ by Eq (16);

 (c) Compute $L_{i}^{\left(j\right)}=L_{i}^{\left(j-1\right)}-\mu^{\left(j-1\right)}\nabla f\left(L_{i}^{\left(j-1\right)}\right)$;

 (d) Update *δ*^(*j*)^ = *cδ*^(*j* − 1)^, 0 < *c* < 1;

 (e) Update *μ*^(*j*)^ = (*δ*^(*j*)^)^2^;

 (f) Output $L_{i}=L_{i}^{\left(j\right)}$ until *j* = *J*.

  **End for**

4.**Inner loop** (solving the **x**-subproblem Eq (13)): for *k* = 1, 2, …, *K* do

 (a) Update *z*^(*k*+1)^ by the Eq (24);

 (b) Update *x*^(*k*+1)^ by the Eq (27);

 (c) Update *d*^(*k*+1)^ by the Eq (23);

 (d) Output *x*^(*s*)^ = *x*^(*k*+1)^ until *k* = *K*.

  **End for**

5. Output the reconstructed image $\hat{x}=x^{\left(s\right)}$ until *s* = *S*.

  **End for**

## Numerical results

Here, we present the experimental results of our approach for CS image reconstruction based on the SRF regularization. We generate the CS measurements by random and pseudo-radial sampling of the Fourier transform coefficients of test images respectively. The number of measurements is *M* = rate*N, where *rate* is the sampling rate. We use peak signal to noise ratio (PSNR) and Structural SIMilarity (SSIM) index [37] as the quantitative measures in our numerical experiments, and the PSNR is defined as

$$\text{PSNR}=20\log_{10}\frac{{\text{MAX}}_{f}}{\sqrt{\text{MSE}}},$$

where MAX_*F*_ is the maximum possible pixel value of the image and MSE is the mean squared error, defined as

$$\text{MSE}=\frac{1}{N}\sum_{j=1}^{N}{\left[f\left(j\right)-g\left(j\right)\right]}^{2},$$

where *f* and *g* are the original image and the restored image, respectively.

In all experiments, the main parameters of the **Algorithm 1** are set as follows: patch size $\sqrt{n}=6$; the number of similar patches for each exemplar patch *m* = 45 (the more similar patches form a more low-rank matrix but lead to high computational complexity); initialize value $\delta^{\left(0\right)}=\bar{\delta }$, where $\bar{\delta }$ is a constant around two times of the largest singular value of initial **L**_*i*_ [30]; the parameter *c* is set as 0.08 experimentally in formula *δ*^(*j*)^ = *cδ*^(*j* − 1)^; the outer loop iteration number *S* = 400 (that is selected based on the convergence rate of **Algorithm 1**). To achieve better performance, we select exemplar patch in each 5 pixels along both horizontal and vertical directions.

**Fig 2:**
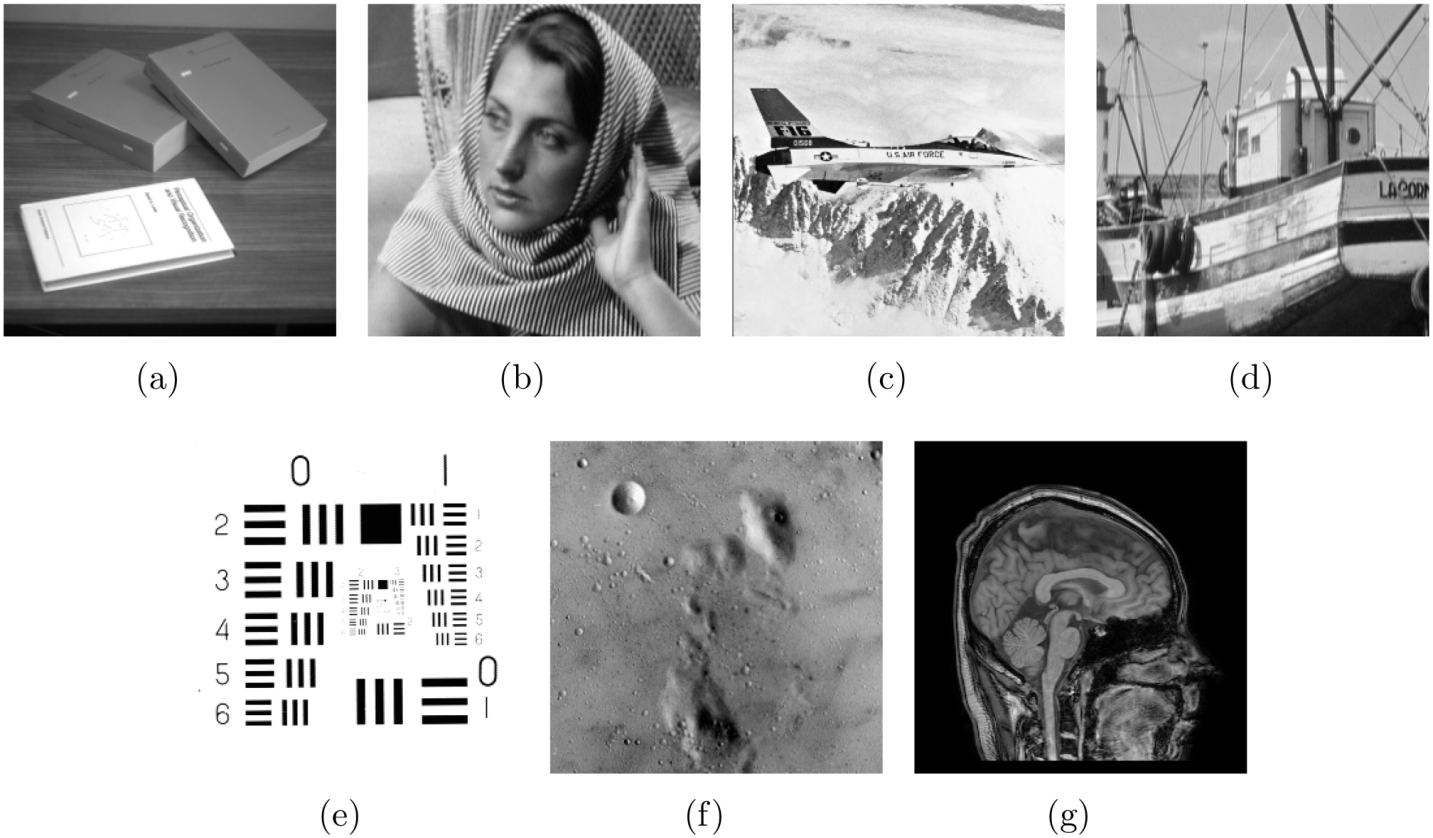
The test images: (a) Book; (b) Barbara; (c) Plane; (d) Boat; (e) Number; (f) Moonscape; (g) Head.

In addition, the total variation (TV) method [38], the BM3D based CS method (BM3D-CS) [16], the nuclear norm method (denoted as NLR-CS-baseline) [22] and the logdet function method (denoted as NLR-CS) [22] for CS image recovery are compared with the proposed SRF approach (called as SRF-CS). The TV method just considered the underlying sparsity of an image, and the BM3D-CS method used the nonlocal sparsity with an outstanding performance. The NLR-CS-baseline method exploited the nuclear norm to replace the rank for solving the rank minimization problem. The NLR-CS approach adopted the logdet function to achieve a more accurate surrogate than the nuclear norm for the rank. The source codes of above methods [16, 22, 38] are publicly downloaded from the authors’s websites. These methods are significantly state-of-the-art CS algorithms for image recovery. We have carefully tuned their parameters to obtain the best results for fair comparison. Both noiseless and noisy experiments are presented to demonstrate the performance of the proposed approach for CS recovery. The test images (256 × 256) are exhibited in Fig 2, where Fig 2(a), 2(c), 2(e) and 2(f) can be publicly downloaded from the test image net http://decsai.ugr.es/cvg/dbimagenes/g256.php, Fig 2(b), 2(d) and 2(g) can be publicly downloaded from the author’s homepage of [22] http://see.xidian.edu.cn/faculty/wsdong/NLR_Exps.htm.

### Noiseless experiments for CS recovery

**Fig 3:**
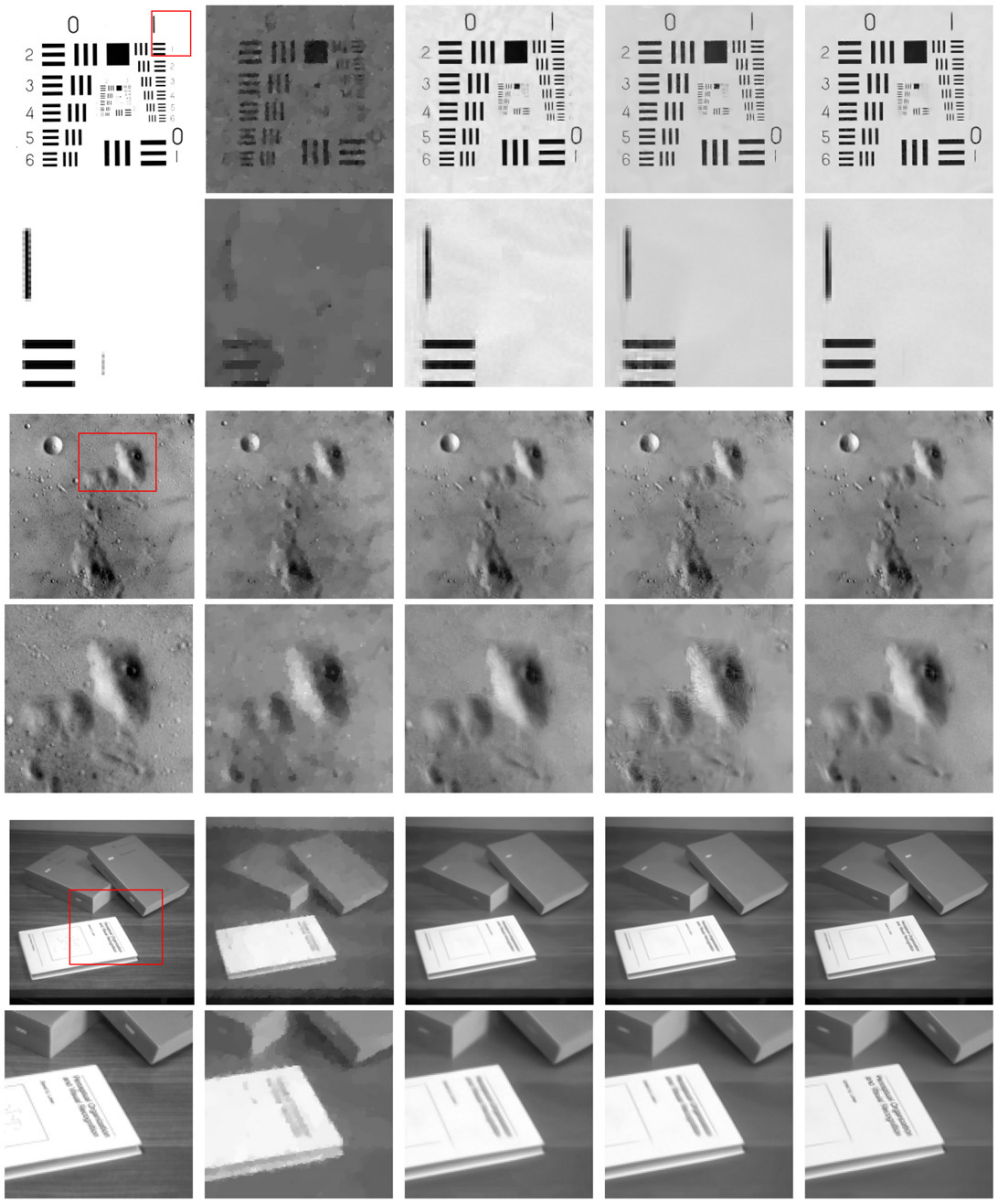
Reconstructed images by randomly sampling. From top to bottom for each column: Number image, close-up of the red rectangle of Number image, Moonscape image, close-up of the red rectangle of Moonscape image, Book image, close-up of the red rectangle of Book image. From left to right for each row: the original image, the TV method, the NLR-CS-baseline method, the NLR-CS method, the proposed method.

We reconstruct the images from less *M* = 0.05*N* and *M* = 0.1*N* measurements by randomly sampling the Fourier transform coefficients of the test images. Fig 3 shows the restored images by TV [38], NLR-CS-baseline [22], NLR-CS [22] and the proposed methods for CS image recovery. The TV method for CS recovery can’t work well due to less measurements. The close-ups of Number image and Book image obtained by the TV method are indistinct and lose much structured information. The NLR-CS-baseline method gives rise to some artificial shadow like fog as shown in the close-up of Number image. It is well known that the NLR-CS method is a very competitive method for CS recovery due to its remarkable performance. For these test images in Fig 3, however, the performance of the NLR-CS method is undesirable. Obviously, our approach achieves the best visual quality and highest PSNR value among all test methods. Noteworthily, the better performance of our approach compared with the NLR-CS-baseline method verifies that the SRF function Eq (8) is a more accurate surrogate for the rank than the nuclear norm. Moreover, Table 1 displays the PSNR values and the SSIM values obtained by all test methods.

**Table 1:** The PSNR (dB) and SSIM values of the restoration results achieved by the TV method [38], the NLR-CS-baseline method [22], the NLR-CS method [22] and the proposed method for noiseless images.

Image	Number of measurements	Method	PSNR (dB)	SSIM
Number	M = 0.05N	TV	24.10	0.6545
		NLR-CS-baseline	26.29	0.8970
		NLR-CS	26.70	0.9407
		proposed	**29.04**	**0.9497**
Moonscape	M = 0.1N	TV	26.41	0.5450
		NLR-CS-baseline	30.19	0.7037
		NLR-CS	29.92	0.7004
		proposed	**30.95**	**0.7143**
Book	M = 0.05N	TV	32.41	0.7304
		NLR-CS-baseline	37.41	0.9371
		NLR-CS	38.44	0.9430
		proposed	**39.77**	**0.9528**

**Fig 4:**
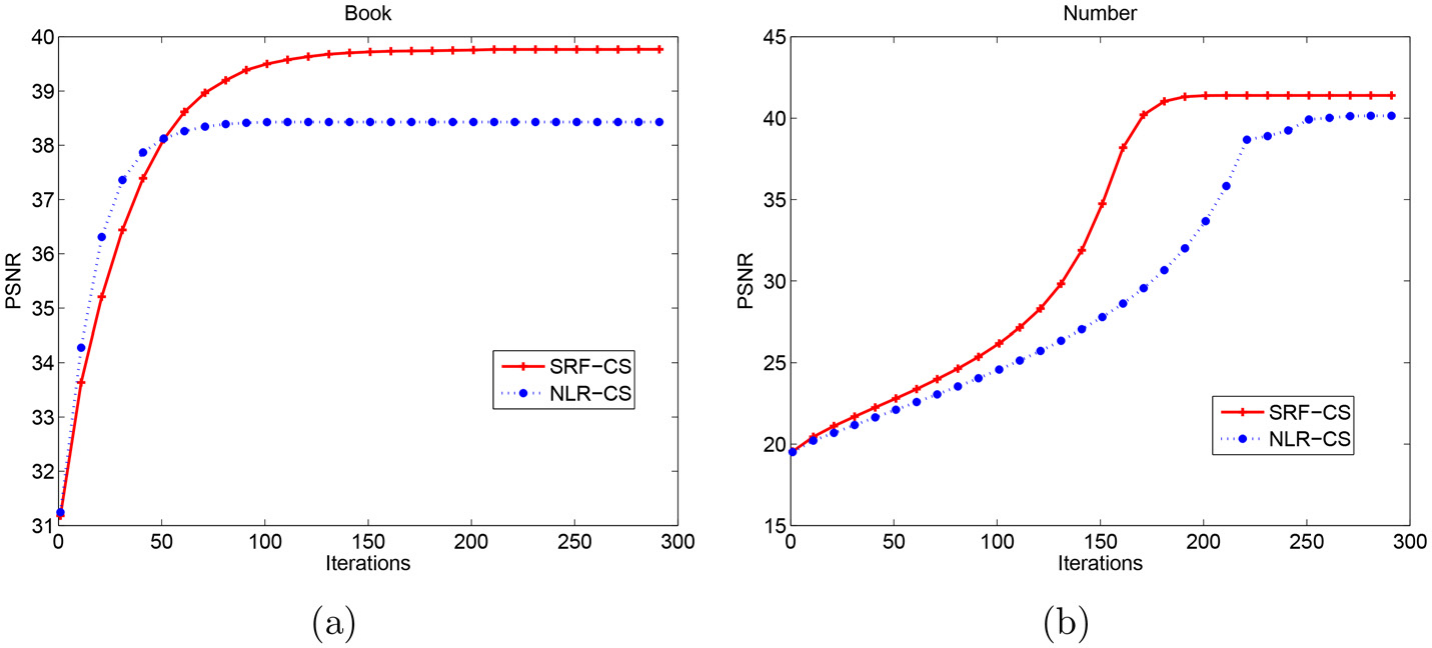
PSNR value vs. Iteration plots for the proposed method and the NLR-CS method. (a) Book image, measures number *M* = 0.05*N* (b) Number image, measurements number *M* = 0.1*N*.

Although the proposed method leads to a non-convex optimization problem, we can see that the proposed method for CS image recovery converges in a few iterations in Fig 4. In the following subsection, we will verify that the proposed method is also robust in a noisy situation.

### Noisy experiments for CS recovery

**Fig 5:**
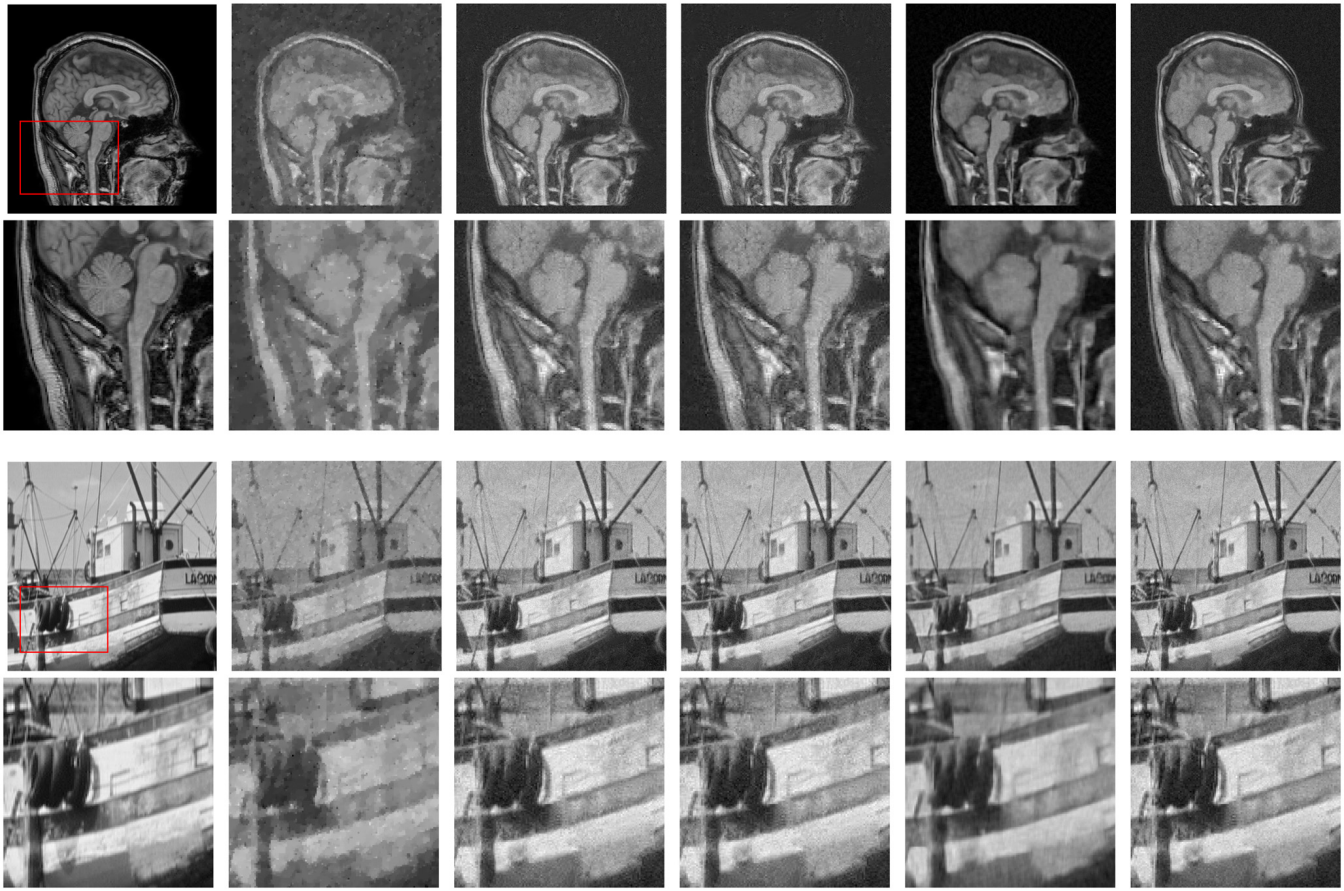
Reconstructed images by randomly sampling. From top to bottom for each column: Head image, close-up of the red rectangle of Head image, Boat image, close-up of the red rectangle of Boat image. From left to right for each row: the original image, the TV method, the NLR-CS-baseline method, the NLR-CS method, the BM3D-CS method, the proposed method.

In the noisy experiments, we add Gaussian noise with 0.05 standard deviation to the original images. Firstly, we recover the Head image from *M* = 0.15*N* measurements and the Boat image from *M* = 0.2*N* measurements by randomly sampling their Fourier transform coefficients respectively. The restoration results by the TV [38], BM3D-CS [16], NLR-CS-baseline [22], NLR-CS [22] and the proposed methods are presented in Fig 5. Clearly, the restored Head image becomes blocky obtained by the TV method, and contains heavy noise obtained by the NLR-CS-baseline method. Meanwhile, some edges in the close-up of the restored Head image achieved by the NLR-CS method are unclear, and the close-up of Head image generated by the BM3D-CS method is blurring. However, compared with other methods, the reconstructed Head image by the proposed approach returns the clearer edges and less noise. For the Boat image, we can see that the proposed approach also outperforms the other test methods with the better visual quality, the higher PSNR and SSIM values. Thus the proposed method with less measurements is robust in noisy situation.

**Fig 6:**
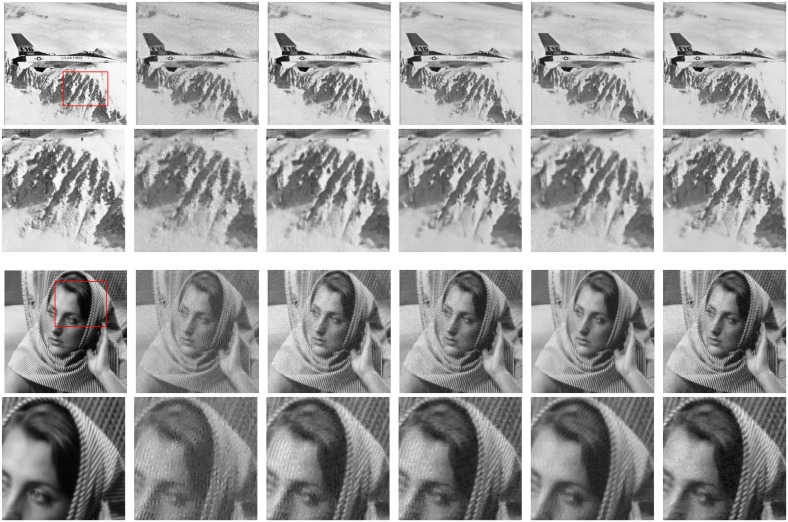
Reconstructed images by pseudo-radial sampling. From top to bottom for each column: Plane image, close-up of the red rectangle of Plane image, Barbara image, close-up of the red rectangle of Barbara image. From left to right for each row: the original image, the TV method, the NLR-CS-baseline method, the NLR-CS method, the BM3D-CS method, the proposed method.

Secondly, we recover the Boy image from *M* = 0.24*N* (65 radial lines) measurements and the Barbara image from *M* = 0.29*N* (80 radial lines) measurements by pseudo-radial sampling their Fourier transform coefficients. The pseudo-radial sampling way generates streaking artifacts leading to more difficult image reconstruction than randomly sampling way. We illustrate the performance results of all the test methods in Fig 6. Visually, the close-ups of Plane image generated by the TV, NLR-CS-baseline, NLR-CS and BM3D-CS methods are blurring. Nevertheless, the close-up of Plane image obtained by our approach is good with less noise and higher PSNR value. The close-ups of Barbara image achieved by the NLR-CS-baseline method and the NLR-CS method create some artificial strips, which do not exist in the original Barbara image. The bad performance may be caused by the pseudo-radial sampling way or noise. The texture of restored Barbara image by BM3D method is lost. However, the proposed approach removes the artifacts and a lot of noise returning a better result, which demonstrates its robustness. In addition, Table 2 displays the PSNR and SSIM values obtained by all test methods. To sum up, the proposed approach outperforms other competing methods for CS image reconstruction by both randomly sampling and pseudo-radial sampling schemes.

**Table 2:** The PSNR (dB) and SSIM values of the restoration results obtained by the TV method [38], the NLR-CS-baseline method [22], the NLR-CS method [22], the BM3D-CS method [16] and the proposed method for noisy images.

Image	Number of measurements	Sampling scheme	Method	PSNR (dB)	SSIM
Head	M = 0.15N	random	TV	23.07	0.5489
			NLR-CS-baseline	27.15	0.6508
			NLR-CS	27.20	0.6515
			BM3D-CS	27.56	**0.7203**
			proposed	**27.76**	0.6615
Boat	M = 0.2N	random	TV	22.90	0.5313
			NLR-CS-baseline	27.60	0.7091
			NLR-CS	27.62	0.7093
			BM3D-CS	27.75	0.7281
			proposed	**27.98**	**0.7397**
Plane	M = 0.2N	pseudo-radial	TV	25.86	0.6410
			NLR-CS-baseline	28.64	0.8657
			NLR-CS	28.98	0.8866
			BM3D-CS	28.55	0.8203
			proposed	**29.38**	**0.9014**
Barbara	M = 0.29N	pseudo-radial	TV	24.09	0.6131
			NLR-CS-baseline	27.79	0.7721
			NLR-CS	28.12	0.7980
			BM3D-CS	28.62	0.8093
			proposed	**29.20**	**0.8231**

## Conclusion

To better exploit the nonlocal sparsity of similar patches and non-convexity of rank minimization, in this paper, we use the non-convex SRF function surrogating the rank as a low-rank regularization for CS image recovery. This SRF function can better approximate the rank than the standard nuclear norm and the logdet function. We propose an efficient algorithm in the framework of alternative minimization method, which divides this CS problem into the SRF minimization subproblem and the least square subproblem. With respect to the minimization subproblem of the SRF function, we adopt the gradient descent method to solve it since it is differentiable. Simultaneously, the clear image is reconstructed by solving the least square subproblem using the ADMM method. Both noiseless and noisy numerical experiments demonstrate that the proposed approach achieves the better performance and vision quality under the lower sampling rate situation. In the future, we would like to explore better surrogates for the rank to improve performance, and solve other practical problems.
